# Real-world comparison of the effects of etanercept and adalimumab on well-being in non-systemic juvenile idiopathic arthritis: a propensity score matched cohort study

**DOI:** 10.1186/s12969-022-00763-x

**Published:** 2022-11-14

**Authors:** Joeri W. van Straalen, Sytze de Roock, Gabriella Giancane, Alessandro Consolaro, Marite Rygg, Ellen B. Nordal, Nadina Rubio-Pérez, Marija Jelusic, Jaime De Inocencio, Jelena Vojinovic, Nico M. Wulffraat, Patricia C. J. Bruijning-Verhagen, Nicolino Ruperto, Joost F. Swart, for the Paediatric Rheumatology International Trials Organisation (PRINTO), Chiara Pallotti, Silvia Scala, Simona Angioloni, Luca Villa

**Affiliations:** 1 Department of Pediatric Immunology and Rheumatology, Wilhelmina Children’s Hospital, University Medical Center Utrecht, P.O. box 85090, 3508 AB Utrecht, The Netherlands; 2 Faculty of Medicine, Utrecht University, Utrecht, the Netherlands; 3 Clinica Pediatrica E Reumatologia, IRCCS Istituto Giannina Gaslini, Genoa, Italy; 4 Dipartimento Di NeuroscienzeRiabilitazioneOftalmologia, Genetica e Scienze Materno-Infantili (DiNOGMI), Università Degli Studi Di Genova, Genoa, Italy; 5 Department of Clinical and Molecular Medicine, Faculty of Medicine and Health Sciences, NTNU - Norwegian University of Science and Technology, Trondheim, Norway; 6 Department of Pediatrics, St. Olavs University Hospital of Trondheim, Trondheim, Norway; 7 Department of Pediatrics, University Hospital of North Norway, Tromsø, Norway; 8 Department of Clinical Medicine, UiT the Arctic University of Norway, Tromsø, Norway; 9 Departamento de Pediatria, Facultad de Medicina, Hospital Universitario “Dr. J. E. González”, Universidad Autónoma de Nuevo León, Monterrey, Mexico; 10 Department of Paediatrics, University of Zagreb School of Medicine, Zagreb, Croatia; 11 Department of Pediatric Rheumatology, University Hospital 12 de Octubre, Madrid, Spain; 12 Department of Pediatric Immunology and Rheumatology, Faculty of Medicine, University of Nis, Nis, Serbia; 13 Department of Pediatric Rheumatology, Clinic of Pediatrics, Clinical Center Nis, Nis, Serbia; 14 Julius Center for Health Sciences and Primary Care, University Medical Center Utrecht, Utrecht, the Netherlands; 15 UOSID Centro Trial, IRCCS Istituto Giannina Gaslini, Genoa, Italy

**Keywords:** Juvenile idiopathic arthritis, Etanercept, Adalimumab, Patient-reported outcomes, Epidemiology, Real-world data, Propensity score analysis

## Abstract

**Background:**

Etanercept (ETN) and adalimumab (ADA) are considered equally effective biologicals in the treatment of arthritis in juvenile idiopathic arthritis (JIA) but no studies have compared their impact on patient-reported well-being. The objective of this study was to determine whether ETN and ADA have a differential effect on patient-reported well-being in non-systemic JIA using real-world data.

**Methods:**

Biological-naive patients without a history of uveitis were selected from the international Pharmachild registry. Patients starting ETN were matched to patients starting ADA based on propensity score and outcomes were collected at time of therapy initiation and 3–12 months afterwards. Primary outcome at follow-up was the improvement in Juvenile Arthritis Multidimensional Assessment Report (JAMAR) visual analogue scale (VAS) well-being score from baseline. Secondary outcomes at follow-up were decrease in active joint count, adverse events and uveitis events. Outcomes were analyzed using linear and logistic mixed effects models.

**Results:**

Out of 158 eligible patients, 45 ETN starters and 45 ADA starters could be propensity score matched resulting in similar VAS well-being scores at baseline. At follow-up, the median improvement in VAS well-being was 2 (interquartile range (IQR): 0.0 – 4.0) and scores were significantly better (*P* = 0.01) for ETN starters (median 0.0, IQR: 0.0 – 1.0) compared to ADA starters (median 1.0, IQR: 0.0 – 3.5). The estimated mean difference in VAS well-being improvement from baseline for ETN versus ADA was 0.89 (95% CI: -0.01 – 1.78; *P* = 0.06). The estimated mean difference in active joint count decrease was -0.36 (95% CI: -1.02 – 0.30; *P* = 0.28) and odds ratio for adverse events was 0.48 (95% CI: 0.16 –1.44; *P* = 0.19). One uveitis event was observed in the ETN group.

**Conclusions:**

Both ETN and ADA improve well-being in non-systemic JIA. Our data might indicate a trend towards a slightly stronger effect for ETN, but larger studies are needed to confirm this given the lack of statistical significance.

**Supplementary Information:**

The online version contains supplementary material available at 10.1186/s12969-022-00763-x.

## Background

Juvenile idiopathic arthritis (JIA) is the most common chronic disease in childhood with a global prevalence varying between 3.8 – 400 per 100,000 [1]. It is not a single disease, but comprises all forms of idiopathic arthritis lasting for more than 6 weeks before the age of 16 [2, 3]. The International League of Associations for Rheumatology (ILAR) has classified seven categories of JIA with distinct clinical and laboratory features [4]. JIA may cause severe disability and a reduced quality of life. Drugs used in the management of JIA are nonsteroidal anti-inflammatory drugs (NSAIDs), intraarticular and systemic glucocorticoids, and conventional synthetic (cs-) and biological (b-) disease-modifying antirheumatic drugs (DMARDs) [5–7]. Due to therapeutic advances in the last two decades, such as the availability of b-DMARDs, disease remission has become a realistic goal for most children with JIA [8].

Two of the most used b-DMARDs in the management of non-systemic arthritis in JIA are the TNF-α inhibitors etanercept (ETN) and adalimumab (ADA). Current treatment recommendations for JIA consider ETN and ADA equal alternatives [5]. Unlike ADA, ETN is not effective against uveitis, an ocular manifestation that affects roughly 1 in every 5 JIA patients [9]. A 2013 study found that ETN is prescribed more often than ADA in daily practice, although JIA patients with a history or at high risk of developing uveitis are more commonly treated with ADA [10]. According to this study, the choice for ETN or ADA treatment primarily depends on physician and patient preferences such as experience with the drug.

While ETN and ADA are considered equally effective in treating arthritis in JIA, no studies have compared their impact on patient-reported evaluation of overall well-being. Patient-reported outcomes such as well-being are important measures in a treat-to-target approach to the management of JIA since they provide a more holistic view of health condition and treatment efficacy than merely disease activity [11–14]. Data on patient well-being after drug therapies might therefore be valuable for making treatment guidelines and recommendations.

The objective of this research was to determine whether ETN and ADA have a differential effect on well-being in patients with non-systemic JIA from the international observational Pharmachild registry [15–18]. We hypothesized that such a difference might be caused by differences in type of side effects, methotrexate (MTX) co-medication (which is more common with ADA in order to prevent anti-drug antibody development) and frequency of the injection ( which is higher for ETN).

## Methods

### Patients

The “Pharmacovigilance in JIA patients treated with biologic agents and/or MTX” (Pharmachild) registry started in 2011 and is currently ongoing. Its primary objective is to assess safety and efficacy of DMARD therapies in patients with JIA. Inclusion criteria are children with JIA as per ILAR classification criteria that are receiving NSAIDs, glucocorticoids, cs-DMARDs or b-DMARDs per physician decision. Currently, patients are enrolled from 85 centers that are part of the Pediatric Rheumatology International Trials Organization (PRINTO) from 31 countries worldwide [19]. Pharmachild consists of patients for whom only retrospective data have been collected at enrolment and patients for whom also prospective data is collected. In brief, Pharmachild collects demographic, clinical and laboratory data, information on drug exposure and adverse events and the cross-culturally adapted version of the Juvenile Arthritis Multidimensional Assessment Report (JAMAR) [20]. The JAMAR assesses patient-reported outcomes in JIA, including functional status, pain, disease activity, health-related quality of life, well-being and satisfaction with disease status [21]. It has been translated into 54 languages and both a parent and child version exist. JAMAR questionnaires in Pharmachild are only available for patients with prospective data. Further details of the Pharmachild registry are available elsewhere [15].

Data of patients with prospective data were extracted on 12 November 2020. For inclusion into the current study, patients or their parents should have completed a “baseline” JAMAR on the day of starting ETN or ADA therapy or at maximum 1 month earlier, provided they had not received any b-DMARD previously. In case both a parent and child JAMAR was completed for the same visit, the child version was selected. In this way, patient-reported outcomes were prioritized over parent-reported outcomes, without excluding information of visits for which only a parent or child JAMAR was available. Other exclusion criteria were systemic JIA, and a history of uveitis. Systemic JIA patients were excluded since this form of JIA is distinct from other subtypes with different clinical features and therapy options [2]. Furthermore, a “follow-up” JAMAR should have been completed 3–12 months after having started ETN or ADA. In case two or more follow-up JAMARs were completed by/for one patient, the JAMAR closest to 6 months after start of ETN or ADA was selected.

### Determinant and outcomes

We compared study outcomes between patients who started ETN versus patients who started ADA. The primary outcome in this study was the improvement in JAMAR visual analogue scale (VAS) well-being score compared to baseline at the follow-up time-point closest to 6 months, with a minimum of 3 and maximum of 12 months. This 21-point VAS score reflects the answer to the following question: “considering all the ways the illness affects you/your child, please evaluate how you/he/she feels at the moment “, and ranges from 0 (very well) to 10 (very poorly). Secondary outcomes were the decrease in active joint count from baseline to follow-up, the number of adverse events reported by the patient or their parent(s) at follow-up and the number of uveitis events that occurred during follow-up.

Other covariates measured at baseline were patient/parent-reported pain, patient/parent-reported evaluation of disease activity, the physician global assessment of disease activity (all measured on a 21-point VAS), the physical and psychosocial domains of the pediatric rheumatology quality of life scale (composite scores of 5 items measured on a 4-point Likert scale), the juvenile arthritis functional score (a composite score of 15 items measured on a 4-point Likert scale), the patient acceptable symptom state (satisfied or not satisfied with current condition) and the Juvenile Arthritis Disease Activity Score (a composite measure consisting of the physician global assessment, VAS well-being, erythrocyte sedimentation rate and the active joint count) [22].

### Propensity score matching

It is difficult to ascertain causal relationships from observational studies due to the lack of randomization typical of clinical trials, which often leads to confounding by indication. This latter term means that certain patients are more likely to receive a treatment of interest than others and therefore run a different risk for the outcome of interest. We addressed this problem by propensity score matching: ETN and ADA starters were matched at baseline on the probability of being prescribed ADA instead of ETN. The following variables at baseline that could play a role in the decision between ETN or ADA therapy [10] were used in a logistic regression model to predict the propensity score: ILAR category of JIA, sex, age, country of medical center, VAS pain, adverse events while on methotrexate therapy and VAS well-being. Before matching the patients, a distribution of propensity scores for ETN and ADA starters was made and patients outside the range of propensity scores that was common for both groups were excluded. This was done in order to eliminate violation of the positivity assumption, which requires that there are no subjects in one treatment group that are not comparable to subjects in the other treatment group based on propensity score [23]. Subsequently, patients were matched 1 to 1 without replacement based on the logit propensity score. For this matching, we used an acceptable distance (i.e. caliper) of 0.2 times the standard deviation of the logit propensity score, as recommended in the literature [24]. Patients with propensity scores outside of the caliper remained unmatched and were excluded for further analysis. After matching, balance in covariates at baseline was assessed by comparing descriptive statistics and by means of the area under the receiver operating characteristic curve (AUC) of the propensity model fitted in the balanced cohort. Several examples of propensity score matching studies exist within the field of rheumatic diseases [25–29], and the authors believe that innovative statistical methods like these are of additive value for evidence-based practice in (pediatric) rheumatology.

### Statistical analysis

Covariates at baseline were compared between ETN and ADA starters using the Mann–Whitney U test, Chi-squared test or Fischer’s exact test. In addition, VAS well-being scores at follow-up, time from baseline measurements to start of the b-DMARD, and time from start of the b-DMARD to follow-up measurements were compared between ETN and ADA starters using the Mann–Whitney U test. Missing outcomes at follow-up were handled by multiple imputation using chained equations. All analyses were run for 20 imputed datasets and the different estimates were combined using the theory of Rubin’s rules, which takes into account both uncertainty from one imputed dataset (within-imputation variability) and uncertainty due to the missing information (between-imputation variability) [30]. Outcomes were analyzed using linear and logistic mixed effects models with a random intercept per treatment center to correct for dependence of observations. We performed an intention-to-treat analysis, that is, patients who started ETN or ADA were analyzed in their respective groups regardless if they stopped or changed initial therapy. The analyses of improvement in VAS well-being and decrease in active joint count (quantitative variables) were adjusted for baseline VAS well-being and baseline active joint count respectively in order to increase statistical power and address the problem of regression to the mean [31]. As a sensitivity analysis, all analyses were repeated for the unmatched cohort of patients meeting the positivity assumption while adjusting for the propensity score (instead of matching). For this analysis, we transformed the propensity score using restricted cubic splines with 4 knots in order to correctly model the relation between this numerical variable and the outcomes of interest [32]. For all analyses, statistical significance was set at *P* < 0.05. All analyses were performed with R version 4.0.0 and the packages rms, mice, lme4, pROC and Matching [33].

## Results

### Matched baseline cohort

**Fig. 1:**
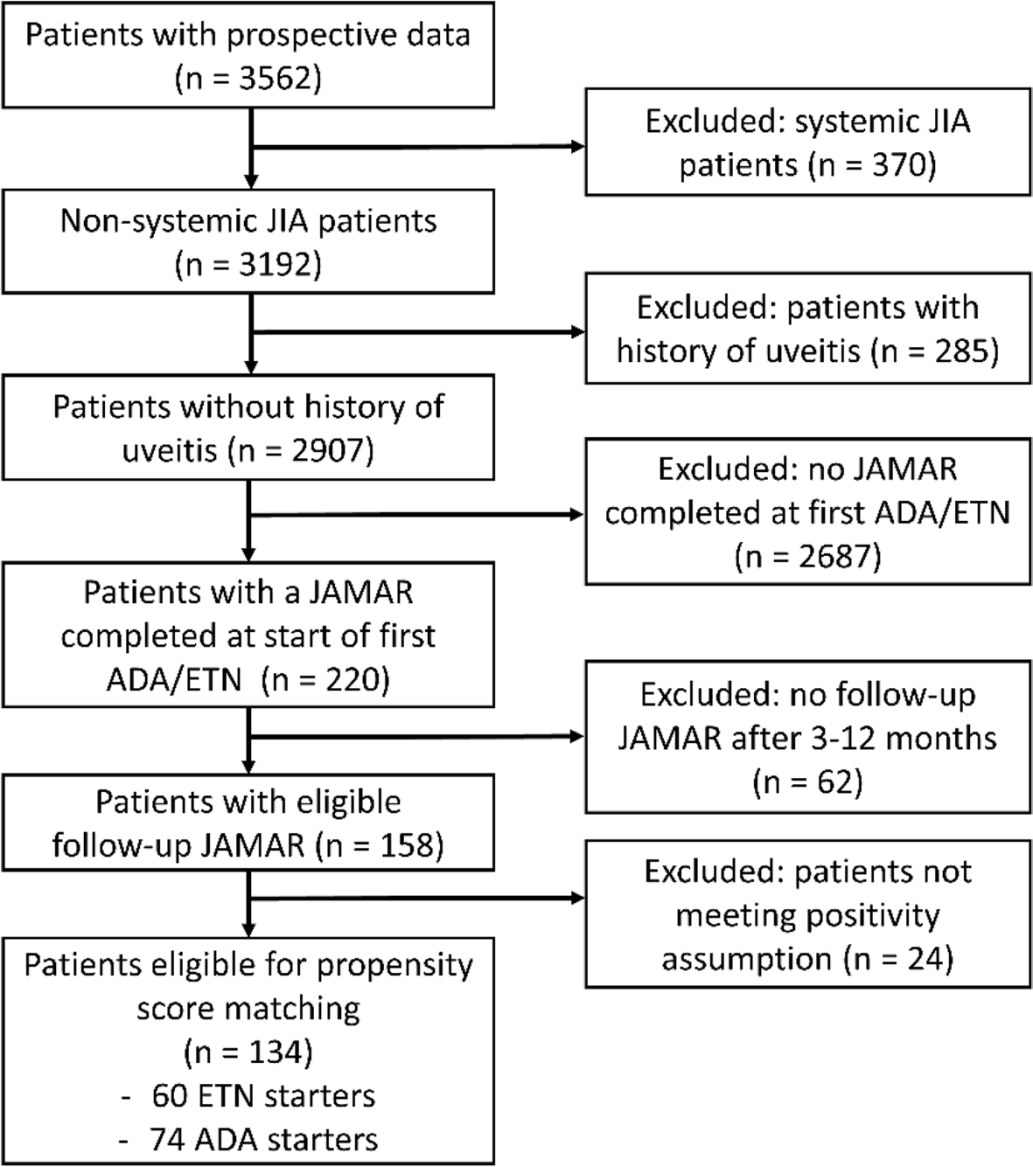
Flowchart of included patients. ADA: adalimumab, ETN: etanercept, JAMAR: juvenile arthritis multidimensional assessment report, JIA: juvenile idiopathic arthritis

As of 12 November 2020, a total of 2,907 non-systemic JIA patients without a history of uveitis were enrolled in the prospective cohort of Pharmachild. Out of these, 158 patients completed a JAMAR at start of ETN/ADA and 3–12 months thereafter (Fig. 1). After calculating propensity scores, another 24 patients who had started ETN had to be excluded because of violation of the positivity assumption. The distribution of propensity scores is provided in an additional figure [see Additional file 1]. Clinical characteristics were similar between included and excluded patients. These are summarized in an additional table [see Additional file 2]. 45/60 ETN starters and 45/74 ADA starters were subsequently matched on propensity score, for whom characteristics used in the propensity score model were similar (Table 1). Further characteristics of the matched patients are summarized in an additional table [see Additional file 3]. The AUC of the propensity score model fitted in the matched baseline cohort was low (0.56, 95% CI: 0.32 – 0.56), indicating a good balance of confounders between ETN and ADA starters. The percentage of patients with a child version JAMAR was comparable for ETN (33.3%) and ADA starters (37.8%). Moreover, the median year of starting ETN (2015, interquartile range (IQR): 2015 – 2016) was close to the median year of starting ADA (2016, IQR: 2015 – 2016). Patients who started ETN had a longer disease duration than patients who started ADA (median 2.9 years versus median 1.5 years, *P* = 0.31). The median VAS pain score in the overall matched cohort was 4.0 (IQR: 1.0 – 6.5), median VAS well-being score was 4.0 (IQR: 1.5 – 6.0) and median active joint count was 3.0 (IQR: 1.0 – 5.8). The median duration from completing a JAMAR to starting a b-DMARD was similar (*P* = 0.15) for ETN (0 days, IQR: 0 – 1) and ADA starters (0 days, IQR: 0 – 7).

**Table 1:** Patient characteristics at baseline

Variable	Cohort before matching (*n* = 134)			Cohort after matching (*n* = 90)		
	ETN starters (*n* = 60)	ADA starters (*n* = 74)	*P*	ETN starters (*n* = 45)	ADA starters (*n* = 45)	*P*
*Demographics*						
Age in years, median (IQR)	8.6 (5.1 – 13.5)	10.7 (6.1 – 14.9)	0.18	8.0 (5.3 – 13.9)	9.8 (5.9 – 14.7)	0.57
Country, n (%)			0.05			1.00
Czech Republic	13 (21.7%)	9 (12.2%)		9 (20.0%)	8 (17.8%)	
France	11 (18.3%)	7 (9.5%)		6 (13.3%)	5 (11.1%)	
Greece	5 (8.3%)	20 (27.0%)		5 (11.1%)	5 (11.1%)	
Italy	19 (31.7%)	20 (27.0%)		16 (35.6%)	18 (40.0%)	
Latvia	0 (0.0%)	1 (1.4%)		0 (0.0%)	0 (0.0%)	
Lithuania	2 (3.3%)	1 (1.4%)		2 (4.4%)	1 (2.2%)	
Netherlands	9 (15.0%)	8 (10.8%)		6 (13.3%)	7 (15.6%)	
Norway	1 (1.7%)	2 (2.7%)		1 (2.2%)	1 (2.2%)	
Poland	0 (0.0%)	1 (1.4%)		0 (0.0%)	0 (0.0%)	
Singapore	0 (0.0%)	2 (2.7%)		0 (0.0%)	0 (0.0%)	
Slovakia	0 (0.0%)	1 (1.4%)		0 (0.0%)	0 (0.0%)	
Spain	0 (0.0%)	2 (2.7%)		0 (0.0%)	0 (0.0%)	
*Clinical characteristics*						
Disease duration in years, median (IQR)	2.4 (1.2 – 5.4)	1.8 (0.8 – 4.1)	0.19	2.9 (1.3 – 5.1)	1.5 (0.8 – 4.4)	0.31
ILAR category, n (%)			0.21			1.00
ERA	7 (11.7%)	17 (23.0%)		6 (13.3%)	7 (15.6%)	
Persistent oligoarthritis	14 (23.3%)	21 (28.4%)		13 (28.9%)	13 (28.9%)	
Extended oligoarthritis	8 (13.3%)	7 (9.5%)		5 (11.1%)	5 (11.1%)	
Polyarthritis RF-	21 (35.0%)	24 (32.4%)		18 (40.0%)	16 (35.6%)	
Polyarthritis RF +	4 (6.7%)	1 (1.4%)		0 (0.0%)	1 (2.2%)	
Psoriatic arthritis	0 (0.0%)	1 (1.4%)		0 (0.0%)	0 (0.0%)	
Undifferentiated arthritis	6 (10.0%)	3 (4.1%)		3 (6.7%)	3 (6.7%)	
Active joint count, median (IQR)	3.0 (2.0 – 7.0)	3.0 (1.0 – 4.8)	0.15	3.0 (1.0 – 6.0)	3.0 (1.0 – 5.0)	0.69
Co-medication, n (%)						
NSAIDs	20 (33.3%)	16 (21.6%)	0.19	16 (34.8%)	10 (22.2%)	0.24
Steroids	9 (15.0%)	12 (16.2%)	1.00	6 (13.0%)	5 (11.1%)	1.00
Synthetic DMARDs	47 (78.3%)	61 (82.4%)	0.71	35 (80.4%)	38 (84.4%)	0.59
*Patient/parent-reported outcomes*						
Adverse events on MTX	20 (33.3%)	27 (36.5%)	0.84	16 (35.6%)	16 (35.6%)	1.00
VAS pain, median (IQR)	4.0 (1.8 – 6.0)	3.3 (0.63 – 6.4)	0.25	4.0 (2.0 – 6.0)	4.5 (1.0 – 6.5)	0.90
VAS well-being, median (IQR)	3.0 (1.5 – 5.1)	4.0 (1.1 – 6.0)	0.74	4.0 (2.0 – 6.0)	4.0 (1.5 – 6.0)	0.78

*ADA* Adalimumab, *ERA* Enthesitis-related arthritis, *ETN* Etanercept, *ILAR* International League of Associations for Rheumatology, *IQR* Interquartile range, *n* Number, *MTX* Methotrexate, *RF* Rheumatoid factor, *VAS* Visual analogue scale

### Follow-up results

**Fig. 2:**
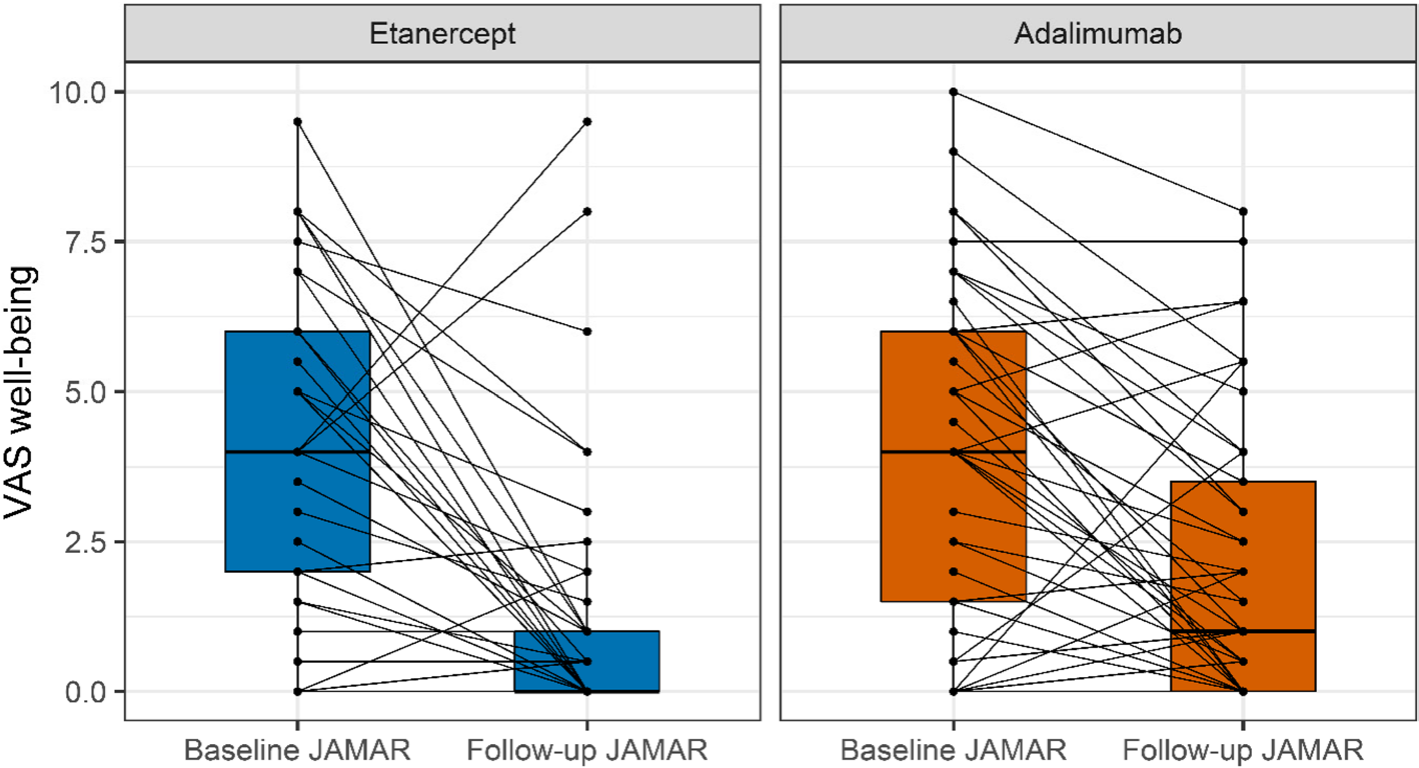
Visual analogue scale (VAS) well-being scores at baseline and follow. Boxplots represent median and interquartile range. Connected dots represent measurements from the same patient. JAMAR: juvenile arthritis multidimensional assessment report

**Fig. 3:**
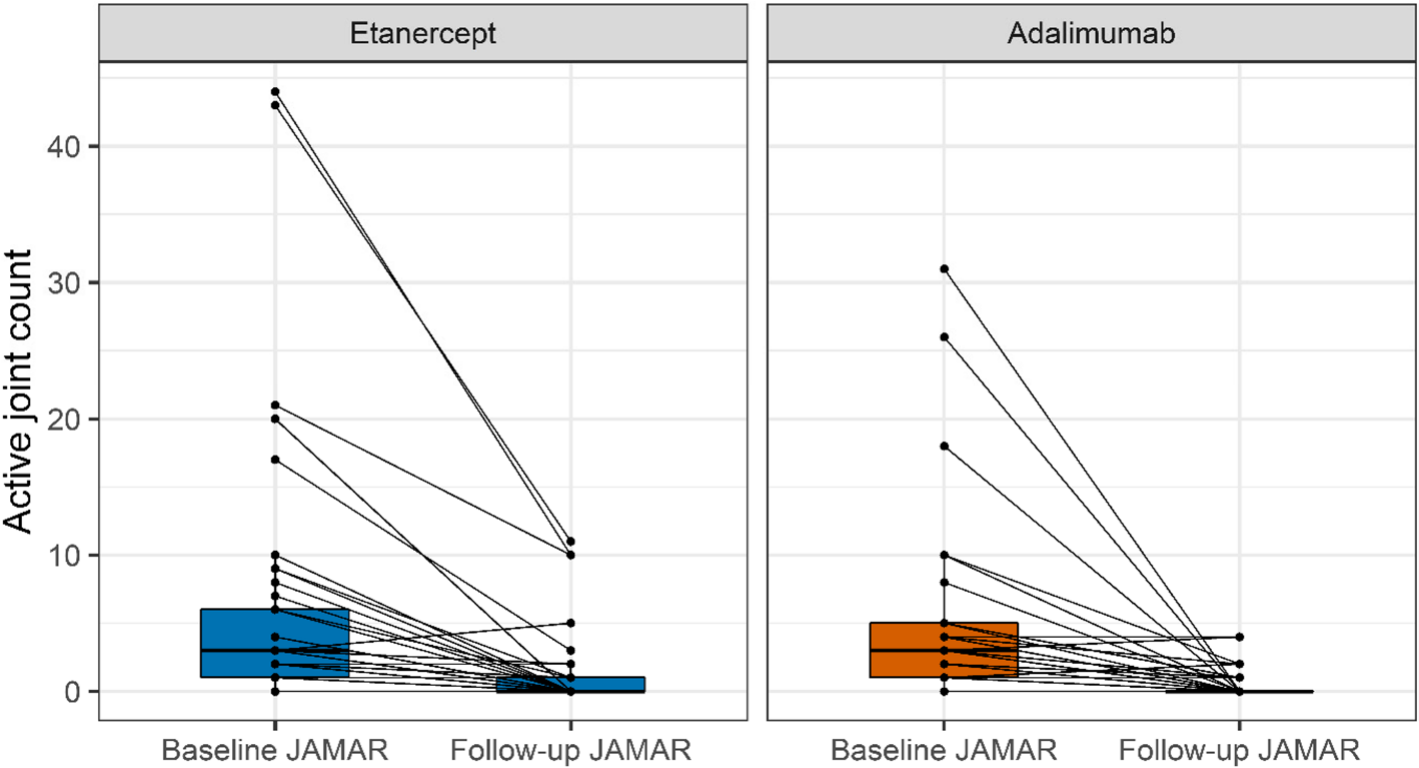
Active joint counts at baseline and follow-up. Boxplots represent median and interquartile range. Connected dots represent measurements from the same patient. JAMAR: juvenile arthritis multidimensional assessment report

The median duration from starting a b-DMARD to completing a follow-up JAMAR was not significantly different (*P* = 0.51) for ETN (183 days, IQR: 168 – 199) and ADA (176 days, IQR: 168 – 195) starters. The distribution of days from starting a b-DMARD to completing a baseline and follow-up JAMAR is provided in an additional figure [see Additional file 4]. At follow up, 42/45 (93%) ETN starters still used ETN and 36/45 (80%) ADA starters still used ADA (*P* = 0.12). VAS well-being scores at follow-up were better (*P* = 0.01) for ETN starters (median 0.0, IQR: 0.0 – 1.0) than ADA starters (median 1.0, IQR: 0.0 – 3.5) (Fig. 2). Nevertheless, a median improvement in VAS well-being of 2 was observed for both ETN (IQR: 0.0 – 5.0) and ADA (IQR: 0.0 – 4.0). The estimated mean difference in VAS well-being improvement for ETN versus ADA starters was 0.89 (95% CI: -0.01 – 1.78) (Table 2). For both groups, 3 patients reported considerable worsening of well-being (VAS well-being increase of ≥ 2). Median active joint count at follow-up was 0 for both ETN and ADA starters (Fig. 3). The estimated mean difference in active joint count decrease for ETN versus ADA starters was -0.36 (95% CI: -1.02 – 0.30). At follow-up, 11 (24.4%) ETN starters and 15 (34.9%) ADA starters reported adverse events. The estimated odds ratio for adverse events between the two groups was 0.48 (95% CI: 0.16 – 1.44). MTX co-medication at follow-up was common for both ETN (60%) and ADA (67%) starters. Patients who started ETN reported more gastric complaints than patients who started ADA, whereas the latter group reported more mood swings and sleep disturbances (Table 3). During follow-up, one event of uveitis occurred in the ETN group.

**Table 2:** Results from follow-up measurements

	ETN starters (*n* = 45)	ADA starters (*n* = 45)	Effect estimate for ETN vs. ADA (95% CI)	*P*-value
Improvement in VAS well-being compared to baseline, median (IQR)	2.0 (0.0 – 5.0)	2.0 (0.0 – 4.0)	0.89 (-0.01 – 1.78)^a^	0.06
Decrease in active joint count compared to baseline, median (IQR)	3 (1 – 6)^b^	2 (1 – 4)	-0.36 (-1.02 – 0.30)^a^	0.28
Adverse events, n (%)	11 (24.4%)	15 (34.9%)^c^	0.48 (0.16 – 1.44)^d^	0.19
Uveitis events, n (%)	1 (2.2%)	0 (0.0%)	-	-

Missing values were handled by multiple imputation

*ADA* Adalimumab, *ETN* Etanercept, *IQR* Interquartile range, *VAS* Visual analogue scale

^a^mean difference as determined from linear mixed effects model

^b^there was one missing observation

^c^there were two missing observations

^d^odds ratio as determined from logistic mixed effects model

**Table 3:** Adverse events reported at follow-up

Adverse event reported, n (%)	ETN starters (*n* = 45)	ADA starters (*n* = 43)	*P*-value
Fever	1 (2.2%)	0 (0.0%)	1
Aphthae	1 (2.2%)	2 (4.7%)	1
Gingivitis	1 (2.2%)	0 (0.0%)	1
Headache	3 (6.7%)	4 (9.3%)	1
Rash	1 (2.2%)	1 (2.3%)	1
Mood swings	1 (2.2%)	5 (11.6%)	0.20
Sleep disturbances	0 (0.0%)	5 (11.6%)	0.06
Gastric complaints	4 (8.9%)	1 (2.3%)	0.36
Nausea	3 (6.7%)	6 (14.0%)	0.48
Vomiting	1 (2.2%)	1 (2.3%)	1
Constipation	1 (2.2%)	0 (0.0%)	1
Injection site reactions	2 (4.4%)	4 (9.3%)	0.68
Dehydration	1 (2.2%)	0 (0.0%	1
Hair loss	0 (0.0%)	2 (4.7%)	0.24
Fatigue	0 (0.0%)	1 (2.3%)	0.49
Urinary incontinence	1 (2.2%)	0 (0.0%)	1
Leukopenia	1 (2.2%)	0 (0.0%)	1

*ADA* Adalimumab, *ETN* Etanercept, *n* Number

### Sensitivity analysis

When analyzing all follow-up measurements of the full unmatched cohort of patients meeting the positivity assumption (*n* = 134), median VAS well-being was 0.5 (IQR: 0.0 – 2.0), median active joint count was 0 (IQR: 0 – 0), 36/132 patients (27.3%) reported adverse events and no additional events of uveitis were reported. Median improvement of VAS well-being and decrease in active joint count from baseline was 2.0 (IQR: 0.0 – 4.3) and 3.0 (IQR: 1.0 – 6.5) for ETN starters and 1.8 (IQR: 0.0 – 4.0) and 2.0 (IQR: 1.0 – 4.0) for ADA starters, respectively. 15 ETN starters (25.0%) and 21 ADA starters (29.2%) reported adverse events. While adjusting for propensity score, the estimated mean difference in VAS well-being improvement for ETN versus ADA starters was 0.70 (95% CI: -0.05 – 1.45). The estimated mean difference in active joint count decrease for ETN versus ADA starters, adjusted for propensity score, was -0.37 (95% CI: -1.27 – 0.52). Finally, the adjusted odds ratio for adverse events between the two groups was 0.45 (95% CI: 0.17 – 1.19). The results from follow-up measurements for the unmatched cohort are summarized in an additional table [see Additional file 5].

## Discussion

In our study, ETN and ADA both improved VAS well-being following 3–12 months of treatment. Analysis of 90 matched patients indicates improvement of well-being may be larger when ETN therapy is prescribed compared to ADA, but results were non-significant. The same conclusions were drawn following a sensitivity analysis in which we used the transformed propensity score for statistical adjustment instead of matching.

Propensity score matching at baseline resulted in overall equally distributed covariates for ETN and ADA starters. However, a difference in median disease duration of over 1 year was observed. It could be that ADA was used earlier in the disease course due to risk of uveitis, which is highest during the first years after onset of arthritis [34]. Nevertheless, when adjusting for baseline disease duration in our analyses, similar results were observed.

We report the first head-to-head comparison of the effects of ETN and ADA on patient-reported evaluation of overall well-being in JIA. Previous studies have reported patient-reported well-being after initiation of ETN or ADA therapy, but did not compare the two drugs [35–37]. In these studies, well-being after anti-TNF therapy improved more compared to the current study, although patients were older, had higher disease activity and could have had systemic arthritis or a history of uveitis. In the current study, VAS well-being scores at follow-up were significantly better for ETN starters compared to ADA starters and the estimated improvement in VAS well-being from baseline was 0.89 points larger for ETN starters compared to ADA starters. The latter difference was however not statistically significant. This may reflect equality between the treatments or a lack of statistical power of our study, given the estimated effect with a significance level of 0.05 was extremely close to statistical significance with a *P*-value of 0.06. A true difference in effect on VAS well-being might be explained by pain caused by ADA injection [10]. Pain on ADA injection used to be associated with a citrate buffer, which was removed from the drug in 2018 [38]. In our study, 89% (40/45) of patients who started ADA did so before 2018. Therefore, it could be that the possible difference in effect on VAS well-being between ETN and ADA is currently smaller than observed in this study.

Similar to the results of our research, previous studies have concluded that ETN and ADA have comparable efficacy in reducing disease activity in JIA [37, 39–42]. However, the evidence from these studies is limited given differences in patient characteristics between the groups of included ETN and ADA users. These differences were mostly observed in uveitis history or earlier b-DMARD use. One study suggested that children younger than 4 years without uveitis show a better response to ETN than ADA [43]. But more research on this subject is required given the risk of de novo uveitis and the fact that ETN and ADA users within this study were also not comparable.

Since the current study did not demonstrate a statistically significant difference in effect on well-being, disease activity and adverse events, presence or risk of uveitis remains the most important factor for physicians to consider when choosing between ETN and ADA. ADA but not ETN is effective against uveitis [9], although development of uveitis has also been reported under ADA therapy [44]. JIA-associated uveitis is extremely rare in patients with systemic arthritis or RF + polyarthritis and occurs most often in ANA positive patients with a young age at JIA onset [17]. Too few uveitis events were observed in the current study to make any comparisons, although the only case of uveitis occurred in the ETN group. Another important factor in choosing between ETN or ADA therapy is possible treatment failure due to development of anti-drug antibodies, which can occur under ADA therapy and can be prevented with MTX co-medication [45]. Adverse events related to MTX are however common and include nausea, gastro-intestinal complaints, mouth ulcers and hepatotoxicity [7]. For these reasons, physicians might opt for ETN instead of ADA therapy, especially in patients with MTX intolerance.

An interesting finding of our study was that well-being considerably worsened during follow-up in 6 patients, although disease activity improved in nearly all patients included in the study. This could possibly be explained by fear of injection, but we could not confirm this hypothesis from JAMARs at follow-up of the concerned patients. Another reason might be chronic pain due to central sensitization, which is not uncommon in JIA [46]. We indeed observed that 4 out of the 6 patients reported a suboptimal VAS pain score and persistent activity or relapse, despite that disease activity, as indicated by physician-reported active and painful joint counts, was absent or minimal. Also, none of these patients developed uveitis. These results show that physician-reported disease activity does not translate directly to well-being in children with JIA.

Our study has limitations. Almost all patients were eventually included from European centers, which might hamper generalization of our results to other settings around the world where b-DMARDs are not widely available [47]. Patients from non-European centers were mostly excluded for not having completed a JAMAR on the day of starting ETN or ADA therapy or at maximum 1 month earlier. Furthermore, the number of patients included in our study was not large enough to draw conclusions about differences in the type of adverse events reported between ETN and ADA starters. Especially considering that a proportion of the reported adverse events were likely caused by MTX co-medication [48], which was common and similar for both ETN and ADA starters at baseline and follow-up. Also, given the observational nature of this study, JAMARs of included patients were not completed at the exact same time points from starting a b-DMARD, further factors associated with uveitis risk such as ANA status and erythrocyte sedimentation rate [49] could not be used in the propensity score model as predictors of ETN or ADA therapy due to missing data, and there is a possibility of unmeasured confounding variables such as the treating physician. The latter could be a confounder given that some physicians might have a preference for ETN or ADA based on previous experiences.

Nonetheless, propensity score matching is a strong method for dealing with bias in (retrospective) observational studies [50]. This method mimics the randomization process of a RCT in the context of a non-interventional study [51]. Indeed, we observed good balance of the many covariates measured in our propensity score-matched cohort. Furthermore, whereas RCTs may prove efficacy of interventions, their results often suffer from limited applicability to clinical practice due to strict inclusion and exclusion criteria. On the other hand, propensity score methods allow for valid comparison of effectiveness of different interventions from “real-world evidence”, which closely resembles the actual clinical practice [52].

To our knowledge, we report the first comparison between similar groups of b-DMARD therapy-naive ETN and ADA starters in JIA, with a focus on patient-reported well-being. Given the scarcity of such data but its value for treatment guidelines and recommendations, more studies on the effects of drugs from the same classes on patient-reported outcomes in JIA should be performed in the future.

## Conclusions

In conclusion, both ETN and ADA resulted in improved well-being in patients with non-systemic JIA. Our data might indicate a trend towards a slightly stronger effect for ETN, but larger studies are needed to confirm this given the lack of statistical significance. Presence or high risk of uveitis and MTX intolerance remain the most important factors to consider when choosing between these two drugs.

## Supplementary Information

**Additional file 1.** Overlapping histograms of propensity score for receiving adalimumab (ADA), *n* = 158.

**Additional file 2.** Characteristics of included and excluded patients.

**Additional file 3.** Extended patient characteristics at baseline.

**Additional file 4.** Stacked histograms of time intervals between start of etanercept/adalimumab therapy and baseline/follow-up measurements.

**Additional file 5.** Results from follow-up measurements for the unmatched cohort.

## Data Availability

All relevant data are reported in the article. Additional details can be provided by the corresponding author upon reasonable request. The Pharmachild registry is registered at Clinicaltrials.gov (NCT01399281) and at the European Network of Centres for Pharmacoepidemiology and Pharmacovigilance (ENCePP; http://www.encepp.eu/encepp/viewResource.htm?id=19362).

## Abbreviations

ADA: Adalimumab
ERA: Enthesitis-related arthritis
ETN: Etanercept
ILAR: International League of Associations for Rheumatology
IQR: Interquartile range
JIA: Juvenile idiopathic arthritis
JAMAR: Juvenile arthritis multidimensional assessment report
n: Number
MTX: Methotrexate
RF: Rheumatoid factor
VAS: Visual analogue scale
