# Supplementary material for: Real-world comparison of the effects of etanercept and adalimumab on well-being in non-systemic juvenile idiopathic arthritis: a propensity score matched cohort study
Source: Pediatr Rheumatol Online J. 2022 Nov 14;20:96. doi: 10.1186/s12969-022-00763-x (PMC9664631; doi:10.1186/s12969-022-00763-x)
Supplement: Supplementary file 1 — Additional file 1. Overlapping histograms of propensity score for receiving adalimumab (ADA), n = 158. [file 12969_2022_763_MOESM1_ESM.docx]

**
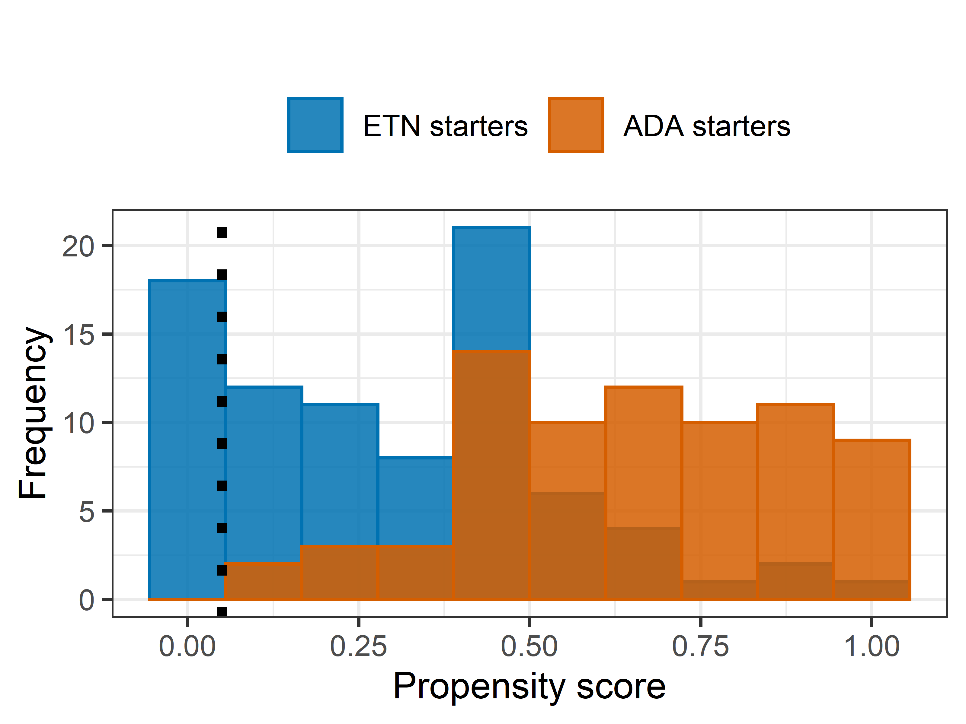

Additional file 1. Overlapping histograms of propensity score for receiving adalimumab (ADA), n = 158.** Vertical black dotted line indicates border of common propensity scores for etanercept (ETN) and ADA starters and patients left from this margin were excluded (n = 24).
