# Supplementary material for: Real-world comparison of the effects of etanercept and adalimumab on well-being in non-systemic juvenile idiopathic arthritis: a propensity score matched cohort study
Source: Pediatr Rheumatol Online J. 2022 Nov 14;20:96. doi: 10.1186/s12969-022-00763-x (PMC9664631; doi:10.1186/s12969-022-00763-x)
Supplement: Supplementary file 2 — Additional file 2. Characteristics of included and excluded patients. [file 12969_2022_763_MOESM2_ESM.docx]

**Additional file 2. Characteristics of included and excluded patients.**

| Variable | Excluded patients (n = 2773) | Included patients (n = 134) |
| --- | --- | --- |
| *Demographics* |  |  |
| Female subjects, n (%) | 1991 (71.8%) | 97 (72.4%) |
| Country, n (%) |  |  |
| Austria | 1 (0.0%) | 0 (0.0%) |
| Brazil | 48 (1.7%) | 0 (0.0%) |
| Croatia | 21 (0.8%) | 0 (0.0%) |
| Czech Republic | 60 (2.2%) | 22 (16.4%) |
| Denmark | 11 (0.4%) | 0 (0.0%) |
| Ecuador | 21 (0.8%) | 0 (0.0%) |
| France | 237 (8.5%) | 18 (13.4%) |
| Greece | 165 (6.0%) | 25 (18.7%) |
| Hungary | 68 (2.5%) | 0 (0.0%) |
| India | 63 (2.3%) | 0 (0.0%) |
| Israel | 23 (0.8%) | 0 (0.0%) |
| Italy | 927 (33.4%) | 39 (29.1%) |
| Latvia | 13 (0.5%) | 1 (0.7%) |
| Libya | 5 (0.2%) | 0 (0.0%) |
| Lithuania | 129 (4.7%) | 3 (2.2%) |
| Mexico | 83 (3.0%) | 0 (0.0%) |
| Netherlands | 175 (6.3%) | 17 (12.7%) |
| Norway | 213 (7.7%) | 3 (2.2%) |
| Poland | 18 (0.6%) | 1 (0.7%) |
| Romania | 78 (2.8%) | 0 (0.0%) |
| Russia | 26 (0.9%) | 0 (0.0%) |
| Saudi Arabia | 35 (1.3%) | 0 (0.0%) |
| Singapore | 37 (1.3%) | 2 (1.5%) |
| Slovakia | 20 (0.7%) | 1 (0.7%) |
| Spain | 295 (10.6%) | 2 (1.5%) |
| Switzerland | 1 (0.0%) | 0 (0.0%) |
| *Clinical characteristics* |  |  |
| Age at JIA onset in years, median (IQR) | 4.5 (2.2 – 9.0) | 5.0 (2.4 – 10.2) |
| ILAR category, n (%) |  |  |
| ERA | 294 (10.6%) | 24 (17.9%) |
| Persistent oligoarthritis | 793 (28.6%) | 35 (26.1%) |
| Extended oligoarthritis | 455 (16.4%) | 15 (11.2%) |
| Polyarthritis RF- | 777 (28.0%) | 45 (33.6%) |
| Polyarthritis RF+ | 131 (4.7%) | 5 (3.7%) |
| Psoriatic arthritis | 100 (3.6%) | 1 (0.7%) |
| Undifferentiated arthritis | 223 (8.0%) | 9 (6.7%) |
| *Immunological markers, n (%)* |  |  |
| ANA positive | 1436 (56.0%) n = 2564 | 73 (56.2%) n = 130 |
| HLA-B27 positive | 387 (26.1%) n = 1482 | 14 (19.4%) n = 72 |
| RF positive | 146 (6.2%) n = 2353 | 5 (4.1%) n = 121 |
| ANA: antinuclear antibodies, ERA: enthesitis-related arthritis, HLA: human leukocyte antigen, ILAR: International League of Associations for Rheumatology, IQR: interquartile range, RF: rheumatoid factor | | |
