# Supplementary material for: Real-world comparison of the effects of etanercept and adalimumab on well-being in non-systemic juvenile idiopathic arthritis: a propensity score matched cohort study
Source: Pediatr Rheumatol Online J. 2022 Nov 14;20:96. doi: 10.1186/s12969-022-00763-x (PMC9664631; doi:10.1186/s12969-022-00763-x)
Supplement: Supplementary file 3 — Additional file 3. Extended patient characteristics at baseline. [file 12969_2022_763_MOESM3_ESM.docx]

**Additional file 3. Extended patient characteristics at baseline.**

| Variable | Cohort before matching (n = 134) | | | Cohort after matching (n = 90) | | |  |  |
| --- | --- | --- | --- | --- | --- | --- | --- | --- |
|  | ETN starters (n = 60) | ADA starters (n = 74) | P | ETN starters (n = 45) | ADA starters (n = 45) | P |  |  |
| Child version of JAMAR | 19 (31.7%) | 32 (43.2%) | 0.23 | 15 (33.6%) | 17 (37.8%) | 0.83 |  |  |
| *Demographics* |  |  |  |  |  |  | |  |
| Female subjects, n (%) | 46 (76.7%) | 51 (68.9%) | 0.42 | 33 (73.3%) | 31 (68.9%) | 0.82 | |  |
| Age in years, median (IQR) | 8.6 (5.1 – 13.5) | 10.7 (6.1 – 14.9) | 0.18 | 8.0  (5.3 – 13.9) | 9.8  (5.9 – 14.7) | 0.57 | |  |
| Country, n (%) |  |  | 0.05 |  |  | 1.00 | |  |
| Czech Republic | 13 (21.7%) | 9 (12.2%) |  | 9 (20.0%) | 8 (17.8%) |  | |  |
| France | 11 (18.3%) | 7 (9.5%) |  | 6 (13.3%) | 5 (11.1%) |  | |  |
| Greece | 5 (8.3%) | 20 (27.0%) |  | 5 (11.1%) | 5 (11.1%) |  | |  |
| Italy | 19 (31.7%) | 20 (27.0%) |  | 16 (35.6%) | 18 (40.0%) |  | |  |
| Latvia | 0 (0.0%) | 1 (1.4%) |  | 0 (0.0%) | 0 (0.0%) |  | |  |
| Lithuania | 2 (3.3%) | 1 (1.4%) |  | 2 (4.4%) | 1 (2.2%) |  | |  |
| Netherlands | 9 (15.0%) | 8 (10.8%) |  | 6 (13.3%) | 7 (15.6%) |  | |  |
| Norway | 1 (1.7%) | 2 (2.7%) |  | 1 (2.2%) | 1 (2.2%) |  | |  |
| Poland | 0 (0.0%) | 1 (1.4%) |  | 0 (0.0%) | 0 (0.0%) |  | |  |
| Singapore | 0 (0.0%) | 2 (2.7%) |  | 0 (0.0%) | 0 (0.0%) |  | |  |
| Slovakia | 0 (0.0%) | 1 (1.4%) |  | 0 (0.0%) | 0 (0.0%) |  | |  |
| Spain | 0 (0.0%) | 2 (2.7%) |  | 0 (0.0%) | 0 (0.0%) |  | |  |
| *Clinical characteristics* |  |  |  |  |  |  | |  |
| Disease duration in years, median (IQR) | 2.4 (1.2 – 5.4) | 1.8 (0.8 – 4.1) | 0.19 | 2.9  (1.3 – 5.1) | 1.5 0.8 – 4.4) | 0.31 | |  |
| ILAR category, n (%) |  |  | 0.21 |  |  | 1.00 | |  |
| ERA | 7 (11.7%) | 17 (23.0%) |  | 6 (13.3%) | 7 (15.6%) |  | |  |
| Persistent oligoarthritis | 14 (23.3%) | 21 (28.4%) |  | 13 (28.9%) | 13 (28.9%) |  | |  |
| Extended oligoarthritis | 8 (13.3%) | 7 (9.5%) |  | 5 (11.1%) | 5 (11.1%) |  | |  |
| Polyarthritis RF- | 21 (35.0%) | 24 (32.4%) |  | 18 (40.0%) | 16 (35.6%) |  | |  |
| Polyarthritis RF+ | 4 (6.7%) | 1 (1.4%) |  | 0 (0.0%) | 1 (2.2%) |  | |  |
| Psoriatic arthritis | 0 (0.0%) | 1 (1.4%) |  | 0 (0.0%) | 0 (0.0%) |  | |  |
| Undifferentiated arthritis | 6 (10.0%) | 3 (4.1%) |  | 3 (6.7%) | 3 (6.7%) |  | |  |
| Co-medication, n (%) |  |  |  |  |  |  | |  |
| NSAIDs | 20 (33.3%) | 16 (21.6%) | 0.19 | 16 (34.8%) | 10 (22.2%) | 0.24 | |  |
| Steroids | 9 (15.0%) | 12 (16.2%) | 1.00 | 6 (13.0%) | 5 (11.1%) | 1.00 | |  |
| Synthetic DMARDs | 47 (78.3%) | 61 (82.4%) | 0.71 | 35 (80.4%) | 38 (84.4%) | 0.59 | |  |
| *Patient/parent-reported outcomes* |  |  |  |  |  |  | |  |
| Adverse events on MTX | 20 (33.3%) | 27 (36.5%) | 0.84 | 16 (35.6%) | 16 (35.6%) | 1.00 | |  |
| VAS pain, median (IQR) | 4.0 (1.8 – 6.0) | 3.3 (0.63 – 6.4) | 0.25 | 4.0  (2.0 – 6.0) | 4.5  (1.0 – 6.5) | 0.90 | |  |
| VAS disease activity, median (IQR) | 4.3 (2.0 – 6.6) | 3.5 (1.0 – 6.0) | 0.25 | 5.0 (2.0 – 7.0) | 4.5 (1.5 – 6.5) | 0.64 | |  |
| VAS well-being,  median (IQR) | 3.0 (1.5 – 5.1) | 4.0 (1.1 – 6.0) | 0.74 | 4.0 (2.0 – 6.0) | 4.0 (1.5 – 6.0) | 0.78 | |  |
| JQL physical health score, median (IQR) | 4.0 (2.0 – 8.3) n = 52 | 3.0 (1.0 – 6.0) n = 69 | 0.16 | 5.0 (2.0 – 8.0) n = 38 | 4.0 (1.0 – 6.0) n = 41 | 0.31 | |  |
| JQL psychosocial health score, median (IQR) | 1.5 (1.0 – 4.0) n = 50 | 2.0 (0.8 – 4.0) n = 68 | 0.79 | 2.0 (1.0 – 4.0) n = 37 | 2.0 (1.0 – 4.0) n = 41 | 0.62 | |  |
| JAFS score, median (IQR) | 3.0 (1.0 – 6.5) n = 51 | 3.0 (0.0 – 6.0) n = 71 | 0.20 | 3.0 (1.0 – 6.0) n = 39 | 3.0 (0.0 – 6.0) n = 42 | 0.70 | |  |
| Patient acceptable symptom state, n (%) | 17 (28.8%) n = 59 | 22 (29.7%) n = 74 | 1.00 | 12 (27.3%) n = 44 | 13 (28.9%)  n = 45 | 1.00 | |  |
| *Disease activity,*  *median (IQR)* |  |  |  |  |  |  | |  |
| Active joint count | 3.0 (2.0 – 7.0) | 3.0 (1.0 – 4.8) | 0.15 | 3.0 (1.0 – 6.0) | 3.0 (1.0 – 5.0) | 0.69 | |  |
| PGA | 4.0 (2.9 – 5.0) | 3.5 (2.5 – 5.5) | 0.98 | 3.5 (3.0 – 4.5) | 3.5 (2.5 – 5.0) | 0.72 | |  |
| JADAS-71 score | 11.8 (8.0 – 16.6) n = 52 | 11.6 (7.0 – 16.0) n = 62 | 0.53 | 11.5 (8.1 – 15.0) n = 39 | 11.7 (6.8 – 15.0) n = 35 | 0.82 | |  |
| ADA: adalimumab, DMARD: disease-modifying antirheumatic drug, ERA: enthesitis-related arthritis, ETN: etanercept, ILAR: International League of Associations for Rheumatology, IQR: interquartile range, JADAS: juvenile arthritis disease activity score, JAFS: juvenile arthritis functional score, JAMAR: juvenile arthritis multidimensional assessment report, JQL: pediatric rheumatology quality of life scale, n: number, MTX: methotrexate, NSAID: non-steroidal anti-inflammatory drug, PGA: physician global assessment, RF: rheumatoid factor, VAS: visual analogue scale | | | | | | | |  |
