# Supplementary material for: Real-world comparison of the effects of etanercept and adalimumab on well-being in non-systemic juvenile idiopathic arthritis: a propensity score matched cohort study
Source: Pediatr Rheumatol Online J. 2022 Nov 14;20:96. doi: 10.1186/s12969-022-00763-x (PMC9664631; doi:10.1186/s12969-022-00763-x)
Supplement: Supplementary file 4 — Additional file 4. Stacked histograms of time intervals between start of etanercept/adalimumab therapy and baseline/follow-up measurements. [file 12969_2022_763_MOESM4_ESM.docx]

**
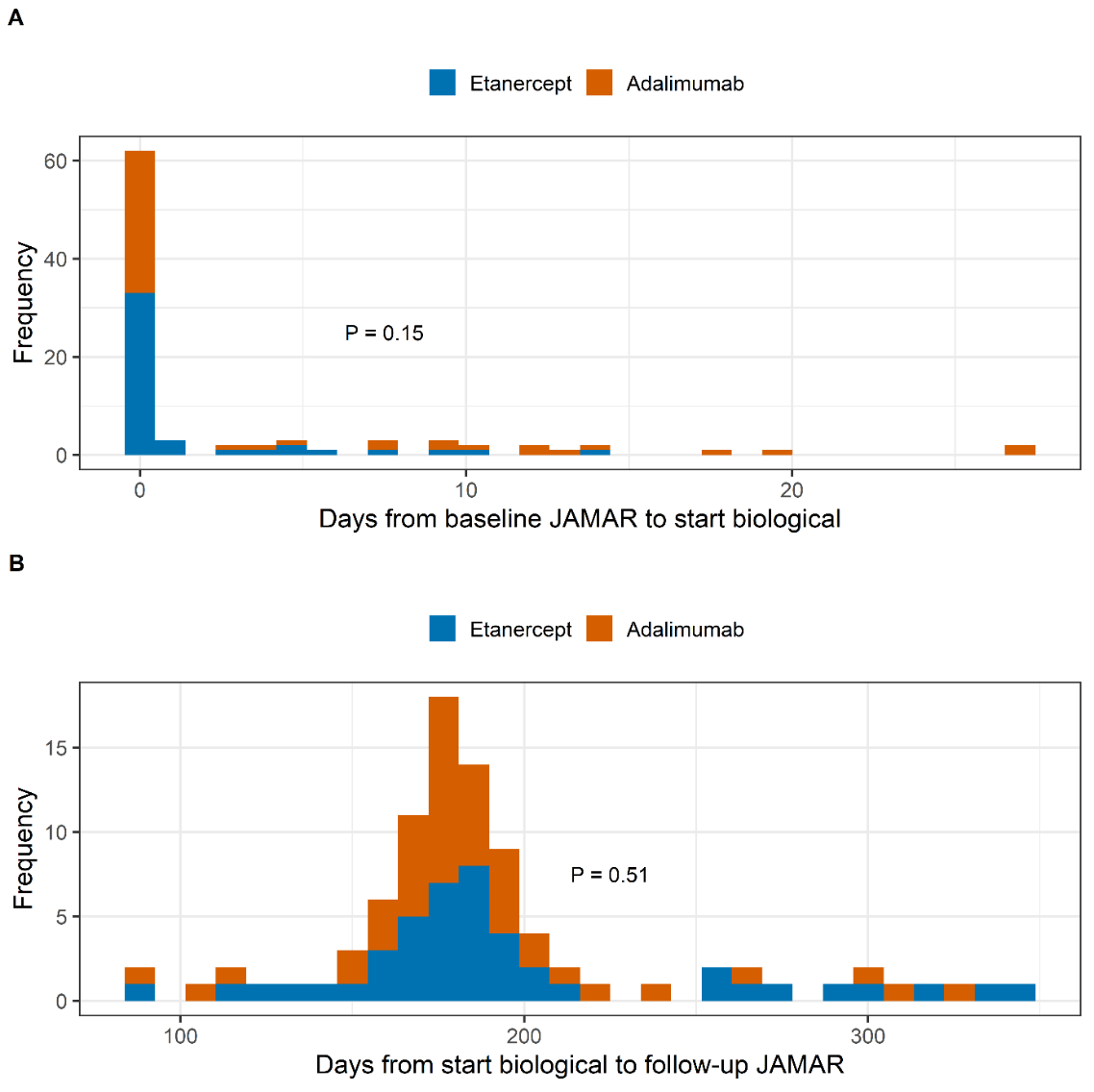
**

**Additional file 4. Stacked histograms of time intervals between start of etanercept/adalimumab therapy and baseline/follow-up measurements.** A: duration from baseline juvenile arthritis multidimensional assessment report (JAMAR) to start biological. B: duration from start biological to follow-up JAMAR.
