# Supplementary material for: Real-world comparison of the effects of etanercept and adalimumab on well-being in non-systemic juvenile idiopathic arthritis: a propensity score matched cohort study
Source: Pediatr Rheumatol Online J. 2022 Nov 14;20:96. doi: 10.1186/s12969-022-00763-x (PMC9664631; doi:10.1186/s12969-022-00763-x)
Supplement: Supplementary file 5 — Additional file 5. Results from follow-up measurements for the unmatched cohort. [file 12969_2022_763_MOESM5_ESM.docx]

**Additional file 5. Results from follow-up measurements for the unmatched cohort.**

|  | ETN starters  (n =60) | ADA starters  (n =74) | PS-adjusted effect estimate for ETN vs. ADA (95% CI) |
| --- | --- | --- | --- |
| Improvement in VAS well-being compared to baseline, median (IQR) | 2.0 (0.0 – 4.3) | 1.8 (0.0 – 4.0) | 0.70 (-0.05 – 1.45)^a^ |
| Decrease in active joint count compared to baseline, median (IQR) | 3.0 (1.0 – 6.5)^b^ | 2.0 (1.0 – 4.0) | -0.37 (-1.27 – 0.52)^a^ |
| Adverse events, n (%) | 15 (25.0%) | 21 (29.2%)^c^ | 0.45 (0.17 – 1.19)^d^ |
| Uveitis events, n (%) | 1 (1.7%) | 0 (0.0%) | - |
| ADA: adalimumab, ETN: etanercept, IQR: interquartile range, PS: propensity score, VAS: visual analogue scale. ^a^mean difference as determined from propensity score-adjusted linear mixed effects model, ^b^there was one missing observation, ^c^there were two missing observations, ^d^odds ratio as determined from propensity score-adjusted logistic mixed effects model. Missing values were handled by multiple imputation. | | | |
